# *Brain Sciences* Special Issue: Insight in the Application of Rehabilitation Devices in Neurological Disease

**DOI:** 10.3390/brainsci15010039

**Published:** 2025-01-02

**Authors:** Maria Cristina De Cola

**Affiliations:** Behavioral and Robotic Neurorehab Unit, IRCCS Centro Neurolesi “Bonino Pulejo”, 98123 Messina, Italy; mariacristina.decola@irccsme.it

It was a great pleasure to be the guest editor for the *Brain Sciences* Special Issue on understanding the use of rehabilitation devices in neurological diseases. This Special Issue consists of a total of seven articles covering a range of topics with the purpose of providing new knowledge and exploring novel interventions that may be used to enhance the rehabilitation process in several neurological diseases. 

Neurorehabilitation is very important because neurological disorders cause significant damage to both cognitive and motor functions and are the leading cause of disease and disability worldwide, according to WHO estimates. Recent technological developments, such as robotic therapy, non-invasive brain stimulation, virtual reality, and telemedicine, support neurorehabilitation therapies and facilitate functional brain recovery. The possibility of providing personalized treatment by designing tasks adapted to the cognitive and physical abilities of the individual increases the validity of interventions and encourages adherence to therapy.

The ability of devices to improve the effectiveness of currently available treatments has been demonstrated by several lines of scientific evidence. About half of the articles in this Special Issue focus on Virtual Reality (VR) applications, both in adult and pediatric rehabilitation. Thus, two studies report interesting results on the use of virtual reality to improve the rehabilitation of children with dyslexia and children with cerebral palsy, showing that VR can improve treatment adherence and minimize symptoms by providing engaging and specific activities for children. Similarly, it appears that transcranial direct current stimulation during VR tasks may improve motor performance in women with fibromyalgia, especially for complex, extensive movements, while VR within an art therapy-based rehabilitation protocol, in which subjects are given the illusion of being able to paint a copy of famous artistic paintings, appears to improve independence in activities of daily living, upper limb muscle strength, and reduce spasticity. In addition, one study on the effectiveness of electrosuit therapy in the clinical treatment of children with cerebral palsy show a discernible improvement in children’s trunk control. The last two studies describe potentially useful tools in the rehabilitation of patients with motor and cognitive disabilities, such as functional magnetic resonance imaging (fMRI), which can be used to decode intentions before the onset of different types of motor imagery, or a free mobile application with psychometric properties. Due to this variety of scenarios, the articles are likely to have a wide readership. 

Taken together, the range of topics covered in this Special Issue is likely to be of interest to both new and experienced researchers working in the field of neurorehabilitation in both children and adults. With this in mind, I would like to thank all of the authors who have contributed to this Special Issue of *Brain Sciences*.

